# Association between the aspartate aminotransferase-to-alanine aminotransferase ratio and the reversion to normoglycemia in people with impaired fasting glucose: a 5-year retrospective cohort study

**DOI:** 10.3389/fendo.2025.1546202

**Published:** 2025-09-25

**Authors:** Kebao Zhang, Lidan Chen, Zhe Deng, Rong Rong, Lifen Xu, Liting Xu, Shuting Zeng, Haofei Hu

**Affiliations:** ^1^ Department of Emergency, The Eighth Affiliated Hospital, Sun Yat-sen University, Shenzhen, Guangdong, China; ^2^ Shenzhen Nanshan Medical Group Headquarters, Shenzhen, Guangdong, China; ^3^ Department of Emergency, Shenzhen Second People’s Hospital, Shenzhen, Guangdong, China; ^4^ Department of Nephrology, Shenzhen Second People’s Hospital, Shenzhen, Guangdong, China

**Keywords:** AST/ALT ratio, impaired fasting glucose, nonlinear relationship, reversion to normoglycemia, inflection point

## Abstract

**Background:**

Studies have shown that the aspartate aminotransferase (AST)/alanine transaminase (ALT) ratio is related to prediabetes, diabetes, and diabetes complications. However, there is limited evidence proving that the AST/ALT ratio is correlated with blood glucose reversal in patients with impaired fasting glucose (IFG). In this study, we analyzed the relationship between the AST/ALT ratio and blood glucose reversal in a large group of Chinese individuals with impaired fasting blood glucose.

**Methods:**

Participants were recruited from the Rich Healthcare Group’s physical examinations from 2010 to 2016. Among them, 11,121 Chinese adults were selected for enrollment in this study. Cox proportional hazards regression was used to identify the association between the AST/ALT ratio and blood glucose reversal to normoglycemia in individuals with IFG. A generalized additive model (GAM) and smooth curve fitting were used to identify a nonlinear relationship between the AST/ALT ratio and blood glucose reversal. In addition, sensitivity analyses and subgroup analyses were used to test the reliability of our study.

**Results:**

The AST/ALT ratio was found to be independently related to blood glucose reversal in pre-diabetic populations of Chinese adults (HR = 1.20, 95%CI = 1.11–1.30, *p* < 0.00001). A nonlinear relationship was found between the AST/ALT ratio and reversion to normoglycemia. On the right side of the inflection point, the AST/ALT ratio was actively related to blood glucose reversal in populations with IFG (HR = 1.37, 95%CI = 1.23–1.52, *p* < 0.0001). However, on the left side of the inflection point, the relationship was not closely related. Sensitivity analyses, competing risk multivariate Cox regression, and subgroup analyses also confirmed the study results.

**Conclusion:**

Our study revealed that the AST/ALT ratio is independently related to reversion to normoglycemia in pre-diabetic Chinese people. The relationship between the AST/ALT ratio and reversion to normoglycemia from IFG is nonlinear. There is a significant positive relationship between the AST/ALT ratio and reversion to normoglycemia when the AST/ALT ratio is >1.13.

## Introduction

Diabetes is a major health concern globally. According to the Global Burden of Disease (GBD) study, the burden of diabetes mellitus (DM) is rising globally. Over 1 million deaths per year are attributed to DM alone, which makes it the ninth leading cause of death (1). Before DM, there is a state known as impaired glucose regulation (also known as prediabetes), which is characterized by an increased glycated hemoglobin (A1C), an impaired fasting glucose (IFG), an impaired glucose tolerance (IGT), or a combination of IFG and IGT (2).

According to the American Diabetes Association (ADA), as updated in 2023, prediabetes is defined as a fasting plasma glucose (FPG) of 5.6–6.9 mmol/L or an IFG (2-h plasma glucose of 7.8–11.0 mmol/L) or A1C of 5.7%–6.4% (3). It is important to note that a prediabetic state is a risk factor for DM. Approximately 5%–10% of patients with a prediabetic state convert to DM each year. In addition, prediabetes itself is a risk factor for developing cardiovascular diseases, such as coronary artery disease and diastolic heart failure (4). Nevertheless, prediabetic patients can also convert back to normoglycemia (2). A previous study found that even a transient reversion to normal glucose levels from prediabetes can reduce the future development of DM; therefore, studying which factors can predict the reversion of prediabetes to normoglycemia is worthwhile (5).

Aspartate aminotransferase (AST) and alanine transaminase (ALT) serve as key biomarkers representing liver function. Research has revealed that the AST/ALT ratio significantly predicts both the likelihood of developing non-alcoholic fatty liver disease (NAFLD) and the risk of developing DM (6, 7). A study conducted in Iran found that the ALT/AST ratio is a risk factor for prediabetes development. Regrettably, the connection between the AST/ALT ratio and the reversion of prediabetes to normoglycemia is hardly understood. Previous studies have found that reversion to normal glucose levels from prediabetes can be attributed to age, gender, race, obesity, lifestyle, baseline A_1_C levels, baseline fasting glucose levels, and pharmacological approaches, among others (8–10). Several studies have elucidated that the AST/ALT ratio is associated with insulin resistance (IR) (11, 12), which can partially explain the relationship between the AST/ALT ratio and DM. We propose the hypothesis that the AST/ALT ratio is positively associated with the reversion to normoglycemia from prediabetes.

## Methods

### Study design

This study is a secondary retrospective study, the aim of which was to identify the association between the AST/ALT ratio and the reversion of IFG to normoglycemia. In this study, we regarded the baseline AST/ALT ratio as the independent variable and the glucose reversion from IFG to normoglycemia during follow-up as the outcome variable.

### Data source

The original data were downloaded freely from the study “Association of body mass index and age with the risk of DM in Chinese adults: a population-based cohort study,” which was uploaded by Chen et al. to the Data Dryad database (www.datadryad.org) and the Dryad Digital Repository (https://datadryad.org/stash/dataset/doi:10.5061%2Fdryad.ft8750v) (13). According to the terms of service stipulated in the Dryad database, researchers are allowed to use the dataset for non-commercial purposes, including sharing, modifying, mixing, or creating derivative works based on the dataset, provided that they cite the author and the source of the data (13).

### Study population

The Rich Healthcare Group recruited participants continuously from 32 locations in 11 cities in China (Hefei, Changzhou, Nantong, Suzhou, Shenzhen, Nanjing, Guangzhou, Shanghai, Chengdu, Wuhan, and Beijing) to minimize selection bias. All procedures involving human subjects were approved by the Clinical Research Ethics Committee of the Rich Healthcare Group. In addition, this study followed the principles outlined in the Helsinki Declaration and used untraceable codes to encode the identity information of participants in order to protect their privacy. Due to the retrospective design and data anonymization, the Institutional Review Committee waived the necessity of informed consent from participants (13, 14).

**Figure 1:**
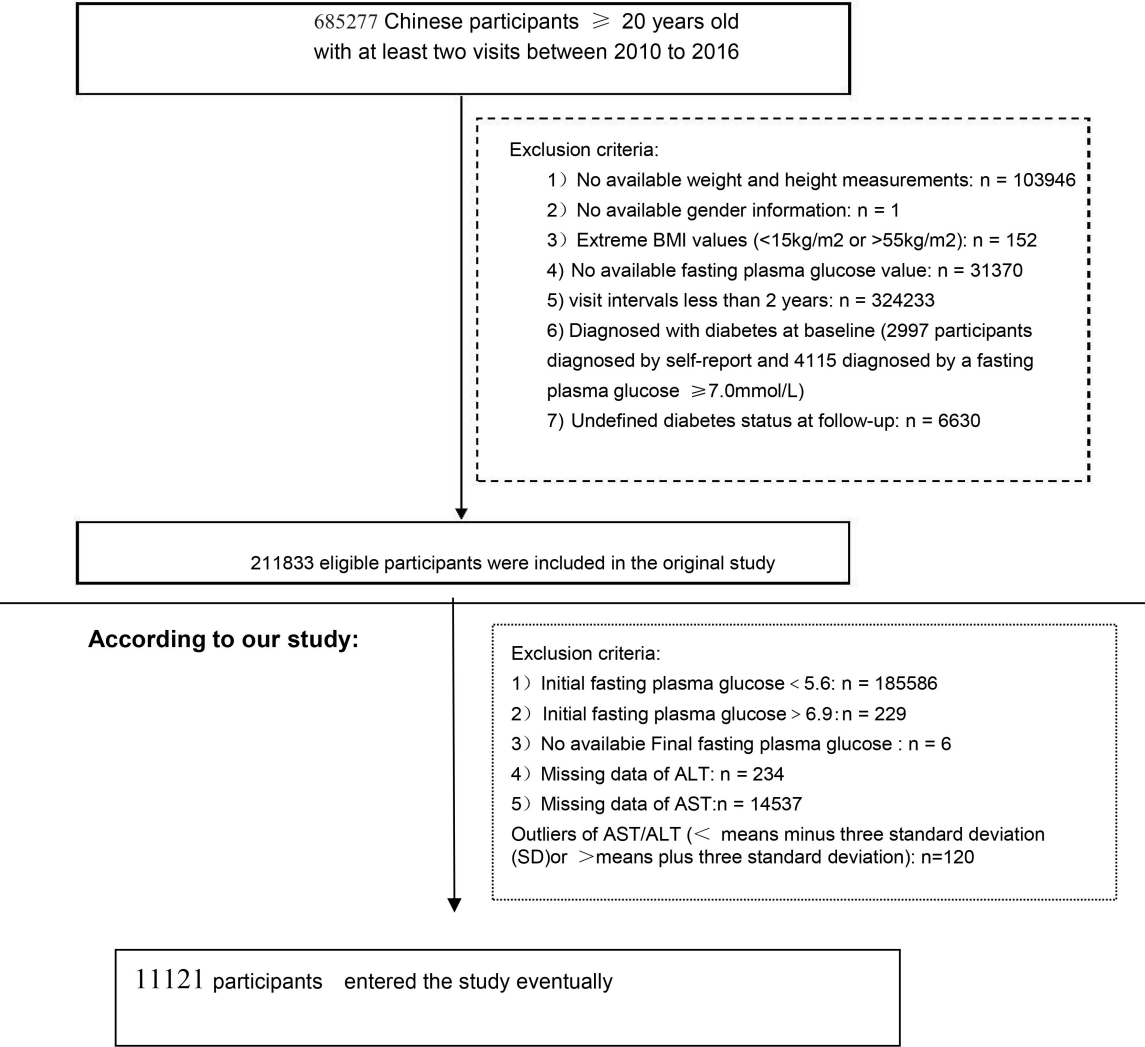
Flowchart of the study participants. Participant selection process. Initially, a total of 211,833 participants were evaluated for eligibility in the original study. After excluding 200,712 individuals, the final analysis consisted of 11,121 subjects.

In the original study, the researchers recruited 685,277 Chinese participants who completed at least a second visit between 2010 and 2016. Ultimately, 211,833 individuals (95,710 women and 116,123 men) were included in the raw analysis based on the following exclusion criteria: 1) missing data on weight, height, gender, and FPG (*n* = 135,317); 2) extreme BMI values (<15 kg/m^2^ or >55 kg/m^2^) (*n* = 152); 3) participant follow-up <2 years (*n* = 324,233); 4) participants diagnosed with diabetes at baseline (4,115 diagnosed by FPG ≥7.0 mmol/L and 2,997 diagnosed by self-report); and 5) participants with an undefined diabetes status at follow-up (*n* = 6,630) (13). In our study, we also excluded the following: 6) initial FPG <5.6 (*n* = 185,586); 7) initial FPG >6.9 (*n* = 229); 8) final FPG not available (*n* = 6); 9) missing ALT data (*n* = 234); 10) missing AST data (*n* = 14,537); and 11) AST/ALT outliers [less than the mean minus 3 standard deviation (SD) or greater than the mean plus 3 SD] (*n* = 120) (15). Finally, a total of 11,121 participants (7,472 men and 3,649 women) were included in the analysis. **Figure 1** shows the details of the participant selection process.

### Variables

#### Aspartate aminotransferase-to-alanine aminotransferase ratio

The baseline AST/ALT ratio was regarded as a continuous variable. The AST/ALT ratio was calculated by dividing the AST (measured in units per liter) by the ALT (measured in units per liter).

#### Impaired fasting glucose

An impaired fasting blood glucose was defined as an FPG value between 5.6 and 6.9 mmol/L.

#### Outcome measures

A successful glucose reversal was defined as no self-reported diabetes events and an FPG level below 5.6 mmol/L during the follow-up (16, 17).

#### Covariates

According to clinical experience and previous research, several variables have been recognized as high-risk factors for diabetes, prediabetes, or diabetes complications (13, 18–22). Therefore, the following covariates were selected for inclusion in this study: 1) continuous variables: blood urea nitrogen (BUN), age, BMI, diastolic blood pressure (DBP), cholesterol, serum creatinine (Scr), low-density lipid cholesterol (LDL-c), triglycerides (TGs), systolic blood pressure (SBP), fasting blood glucose (FPG), and high-density lipid cholesterol (HDL-c). 2) Categorical variables: family history of diabetes, smoking and drinking status, and gender.

### Data collection

The height, weight, and blood pressure of the participants were measured by professional staff. To ensure the accuracy of the data, participants were required to wear lightweight clothing and to remove their shoes before height and weight measurements were taken. Blood pressure was measured using a standard mercury sphygmomanometer. Before measurement, participants were required to stay in a lying position quietly for 5–10 min. BMI was calculated by dividing the weight (in kilograms) by the square of their height (in meters), with a height measurement accuracy of 0.1 cm and a weight measurement accuracy of 0.1 kg. Family history of diabetes, lifestyle data, personal medical history, and demographic characteristics were collected using a survey questionnaire. Clinical variables, such as the BUN, FPG, ALT, HDL-c, TC, LDL-c, TG, AST, and Scr, were obtained with an autoanalyzer (Beckman 5800) after fasting for at least 10 h (13).

### Missing data processing

Some of the variables had missing data in this study. For example, five participants (0.045%) lacked DBP and SBP data, 88 individuals (0.79%) had missing cholesterol and TG data, and 520 participants (4.68%) lost their Scr data. At the same time, a number of variables lacked a significant proportion of data. There were missing data for LDL-c, HDL-c, drinking status, and smoking status in 31.99%, 36.80%, 69.65%, and 69.65% of participants, respectively. We adopted the Multiple Imputation by Chained Equations (MICE) method to handle the missing data and maximize the use of the participants’ data (23). Ultimately, the variables contained in this article include TG, age, AST, DBP, FPG, HDL-c, gender, BMI, SBP, smoking and drinking status, LDL-c, ALT, BUN, Scr, and family history of diabetes. Missing data were handled based on the assumption of missing at random (MAR) (24).

### Statistical analysis

The participants were classified into quartiles according to their AST/ALT ratios: Q1 < 0.81; 0.81 ≤ Q2 < 1.08; 1.08 ≤ Q3 < 1.40; and 1.40 ≤ Q4. Continuous variables were divided into normally distributed values and skewed values, which were described using the mean standard deviation (SD), and median. Categorical values were described by frequencies and percentages. To evaluate the differences between the AST/ALT ratio groups, one-way ANOVA tests were used to analyze the normally distributed values, while chi-square tests and the Kruskal–Wallis *H* test were used to analyze the categorical values and skewed values, respectively. The Kaplan–Meier method was employed to calculate survival estimates and the time-to-event variables. In addition, the log-rank test was used to compare the likelihood of reverting from IFG to normoglycemia among the AST/ALT ratio groups.

The variance inflation factor (VIF) was used to evaluate the likelihood of covariate collinearity, calculated as VIF = 1/(1 − *R* ^2^). *R* was obtained from the linear regression equation (25). The studied variable was treated as the dependent variable, while the other variables were regarded as independent variables in each regression analysis. As a result, the VIF of the variable greater than 5 indicated collinearity between the variables, which meant that this variable was excluded when analyzing the multiple regression model. As shown in **Supplementary Table S1**, the VIF of cholesterol was 6. This variable was excluded in the analysis of the multiple regression model (Attachment 1: **Supplementary Table S1**).

Three different models were constructed using multivariate Cox proportional hazards regression analysis and univariate analysis to determine the relationship between the AST/ALT ratio and the probability of reverting to normoglycemia from IFG. Model I was not adjusted for any covariates, while model II was only adjusted for demographic characteristics such as smoking and drinking status, SBP, gender, DBP, BMI, family history of diabetes, and age. Model III was adjusted for all of the covariates listed in **Table 1**, including Scr, smoking status, age, SBP, HDL-c, gender, TG, drinking status, BMI, FPG, LDL-c, DBP, and family history of diabetes. The article provided hazard ratios (HRs) and their corresponding 95% confidence intervals (CIs). We adjusted for confounding variables in this article based on clinical knowledge and published reports (20, 21, 26). According to the commonality screening results, there was no collinearity issue between any of the variables (Additional file 1: **Supplementary Table S1**).

**Table 1:** Baseline characteristics of the participants.

AST/ALT	Q1 (<0.81)	Q2 (0.81–1.08)	Q3 (1.08–1.40)	Q4 (>1.40)	*P*
No. of participants	2,780	2,780	2,747	2,814	
Age (years)	44.28 ± 11.16	49.68 ± 12.84	52.22 ± 14.24	53.40 ± 15.52	<0.001
BMI (kg/m^2^)	26.49 ± 3.15	25.33 ± 3.13	24.33 ± 3.10	23.26 ± 3.08	<0.001
SBP (mmHg)	128.42 ± 16.01	127.58 ± 17.27	127.75 ± 18.43	126.82 ± 18.91	0.009
DBP (mmHg)	80.35 ± 10.71	78.92 ± 10.88	78.03 ± 11.17	76.55 ± 11.43	<0.001
FPG (mmol/L)	5.97 ± 0.32	5.96 ± 0.32	5.95 ± 0.31	5.92 ± 0.30	<0.001
Cholesterol (mmol/L)	5.10 ± 0.96	4.97 ± 0.93	4.96 ± 0.94	4.92 ± 0.95	<0.001
Triglycerides (mmol/L)	1.80 (1.22–2.69)	1.56 (1.05–2.30)	1.30 (0.90–1.90)	1.08 (0.77–1.59)	<0.001
HDL-c (mmol/L)	1.27 ± 0.28	1.32 ± 0.33	1.36 ± 0.29	1.43 ± 0.29	<0.001
LDL-c (mmol/L)	2.97 ± 0.73	2.88 ± 0.69	2.88 ± 0.70	2.83 ± 0.70	<0.001
ALT (U/L)	42.00 (32.40–60.12)	25.40 (21.00–31.00)	19.00 (15.60–22.50)	13.10 (11.00–16.00)	<0.001
AST (U/L)	31.37 ± 14.88	25.64 ± 9.98	24.20 ± 8.50	24.14 ± 10.44	<0.001
AST/ALT	0.65 ± 0.11	0.94 ± 0.08	1.22 ± 0.09	1.73 ± 0.28	<0.001
BUN (mmol/L)	5.07 ± 1.19	5.12 ± 1.22	5.05 ± 1.25	5.02 ± 1.39	0.030
Scr (μmol/L)	77.14 ± 14.17	75.48 ± 15.79	73.07 ± 16.12	70.71 ± 17.40	<0.001
Gender					<0.001
Men	2,407 (86.58%)	2,068 (74.39%)	1,679 (61.12%)	1,318 (46.84%)	
Women	373 (13.42%)	712 (25.61%)	1,068 (38.88%)	1,496 (53.16%)	
Smoking status					<0.001
Current smoker	542 (19.50%)	449 (16.15%)	336 (12.23%)	296 (10.52%)	
Ever smoker	119 (4.28%)	109 (3.92%)	86 (3.13%)	64 (2.27%)	
Never smoker	2,119 (76.22%)	2,222 (79.93%)	2,325 (84.64%)	2,454 (87.21%)	
Drinking status					<0.001
Current drinker	133 (4.78%)	145 (5.22%)	116 (4.22%)	87 (3.09%)	
Ever drinker	640 (23.02%)	549 (19.75%)	425 (15.47%)	337 (11.98%)	
Never drinker	2,007 (72.19%)	2,086 (75.04%)	2,206 (80.31%)	2,390 (84.93%)	
Family history of diabetes					0.010
No	2,692 (96.83%)	2,718 (97.77%)	2,696 (98.14%)	2,752 (97.80%)	
Yes	88 (3.17%)	62 (2.23%)	51 (1.86%)	62 (2.20%)	

Values are *n* (%), mean ± SD, or median (quartiles).

*BMI*, body mass index; *LDL-c*, low-density lipoprotein cholesterol; *FPG*, fasting plasma glucose; *AST*, aspartate aminotransferase; *DBP*, diastolic blood pressure; *Scr*, serum creatinine; *SBP*, systolic blood pressure; *BUN*, blood urea nitrogen; *ALT*, alanine aminotransferase; *HDL-c*, high-density lipoprotein cholesterol; *AST/ALT ratio*, aspartate aminotransferase/alanine aminotransferase ratio.

A Cox proportional hazards regression model with smooth curve fitting and a cubic spline function were used to investigate a potential nonlinear correlation between the AST/ALT ratio and reversion to normoglycemia in participants with IFG. This statistical method enabled handling any nonlinear problems present in the data. When nonlinearity was detected between data, recursive algorithms were used to identify the inflection points. Subsequently, a two-stage Cox proportional risk regression model was employed on both sides of the inflection point, and a log-likelihood ratio test was also conducted to determine the most appropriate model for evaluating the relationship between the AST/ALT ratio and the reversion to normoglycemia (27).

Considering that people diagnosed with diabetes during the follow-up are unlikely to return to normal blood glucose levels, it was necessary to exclude these participants to avoid affecting the probability of blood glucose reversal in patients with IFG (28, 29). Therefore, the method described by Fine and Gray was used to achieve multivariate Cox regression of competing risks (29, 30). In this approach, developing diabetes is considered to be a competing risk to returning to normal glycemic levels.

We conducted a stratified Cox proportional hazards model based on family history of diabetes, TG, gender, age, BMI, DBP, drinking status, SBP, smoking status, and FPG to perform subgroup analyses. First, categorical variables were established using clinically significant critical points, such as DBP (≥90 and <90 mmHg), BMI (≥28, ≥24 to 28, ≥18.5 to <24, and <18.5 kg/m^2^), TG (≥1.7 and <1.7 mmol/L), age (≥45 and < 45 years), SBP (≥140 and <140 mmHg), and FPG (≥6.1 and <6.1 mmol/L) (31–34). Second, each stratification was adjusted based on all other factors (Scr, TG, age, BUN, ALT, family history of diabetes, DBP, AST, gender, FPG, BMI, SBP, smoking and drinking status, and LDL-c), including the stratification factors themselves. Finally, the models with and without interaction terms were compared to evaluate the interactions by conducting a likelihood ratio test (35, 36).

In addition, a range of sensitivity analyses were conducted to determine the reliability of the results. First, the AST/ALT ratios were divided into quartiles and then converted into a continuous variable, with the *p*-value of the trend calculated. This also helped in identifying a potential nonlinear relationship. As is widely known, individuals who smoke, consume alcohol, or have a family history of diabetes are at an increased risk of developing diabetes (37, 38). Therefore, we excluded individuals with a history of smoking, alcohol consumption, or family history of diabetes when exploring the association between the AST/ALT ratio and the reversion to normoglycemia in individuals with IFG in the additional sensitivity analyses. Furthermore, due to missing data on smoking and alcohol consumption in variables exceeding 70%, smoking and drinking status were eliminated as covariates in the multivariate model, as these might not have any impact on model adjustment. In addition, to ensure the consistency of the research results, a generalized additive model (GAM) was also adopted to incorporate continuous covariates as curves into model IV (39). Moreover, *E*-values were calculated to evaluate the potential impact of the association between the AST/ALT ratio and the reversion to normoglycemia (40). This method provided more evidence of the reliability of the results.

Furthermore, the area under the receiver operating characteristic (ROC) curve (AUC) and its 95% CIs were used to determine the accuracy of the serum uric acid AST/ALT ratio. The ROC curve was also utilized to analyze the performance of the AST/ALT ratio and to determine the optimal cutoff value with the Youden index. At the same time, the corresponding specificity (SP), sensitivity (SE), negative predictive value (NPV), and positive predictive value (PPV) were also calculated.

Data analysis was conducted through two statistical software packages: R Foundation (http://www.R-project.org) and Empower Stats (X&Y Solutions, Inc., Boston, MA, USA; http://www.empowerstats.com). All statistical tests were conducted using a double-sided test, and results with a significance level of *p* < 0.05 were considered statistically significant.

## Results

### Baseline characteristics of the participants

**Figure 2:**
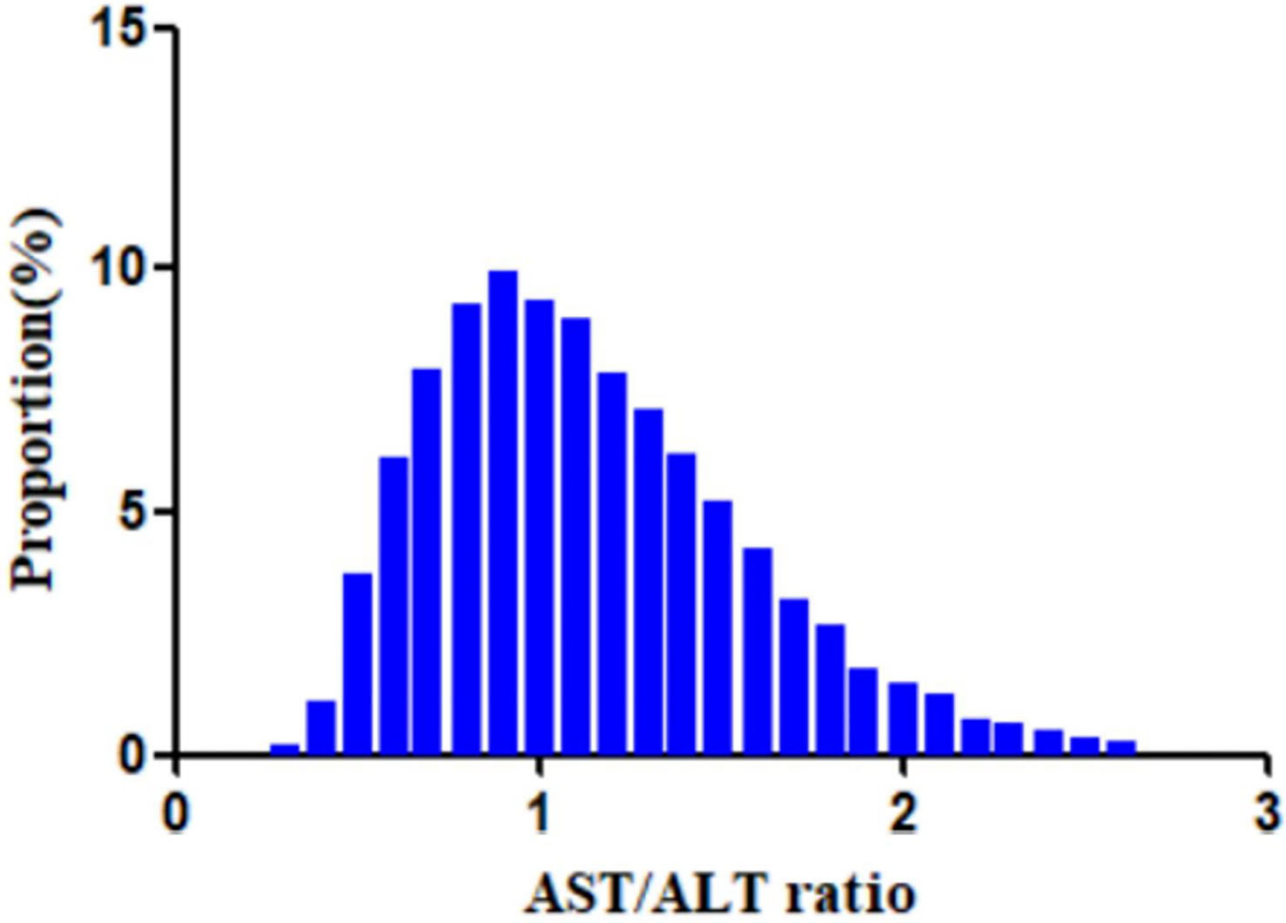
Distribution of the aminotransferase (AST)/alanine transaminase (ALT) ratio. A normal distribution of the aspartate aminotransferase (AST)/alanine transaminase (ALT) ratio is shown, ranging from 0.29 to 2.65, with a mean value of 1.14.

**Table 1** shows the baseline characteristics of the participants included in the study. A total of 11,121 participants were included, 67.2% of whom were men. The mean age of the population was 49.9 ± 14.0 years. The baseline AST/ALT ratio had an average of 1.1 ± 0.4. In the considered population, 41.4% of patients with IFG reverted to normoglycemia during the average follow-up years (3 years). We stratified the AST/ALT ratios into four groups: Q1 < 0.81; 0.81 ≤ Q2 < 1.08; 1.08 ≤ Q3 < 1.40; and 1.40 ≤ Q4. All of the covariates presented in **Table 1** showed statistical significance between the different quartiles of the AST/ALT ratios (*p* < 0.05). Between Q4 (≥1.4) and Q1 (<0.81), significant increments in age, HDL-c, AST/ALT, female gender, and never smoker status were discovered. However, there were opposite trends for BMI, cholesterol, TG, ALT, Scr, male gender, current smoker, ever smoker, and ever drinker among the covariates. **Figure 2** illustrates the distribution of the AST/ALT ratios, which showed a normal distribution with a range of values from 0.29 to 2.65 and a mean value of 1.14.

### Reversion from IFG to normoglycemia

**Figure 3:**
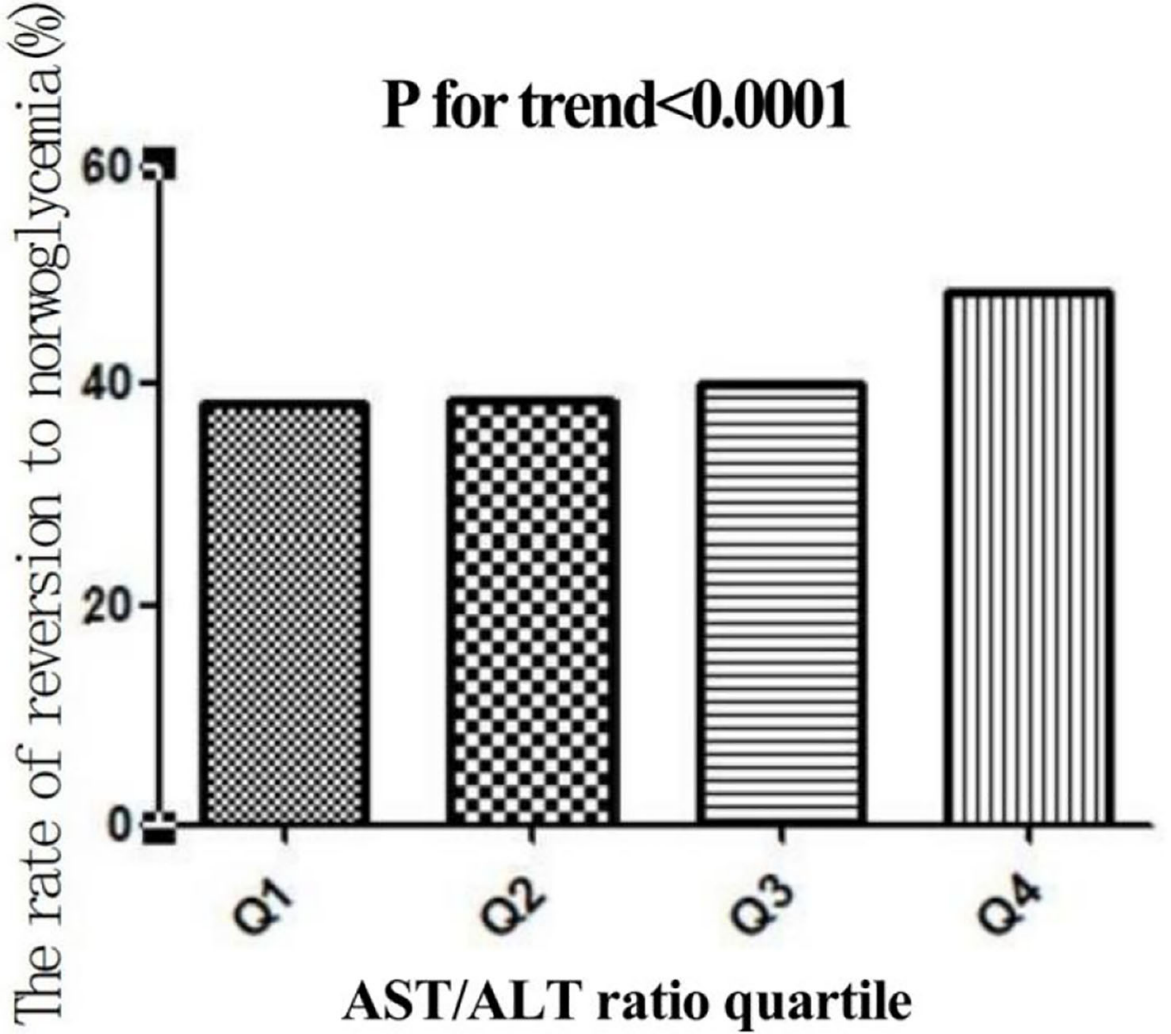
Rate of reversion to normoglycemia in individuals with prediabetes stratified by the quartiles of the aspartate aminotransferase (AST)/alanine transaminase (ALT) ratio. Participants with higher AST/ALT ratios showed higher rates of reversion to normoglycemia (*p* < 0.0001 for trend).

**Figure 4:**
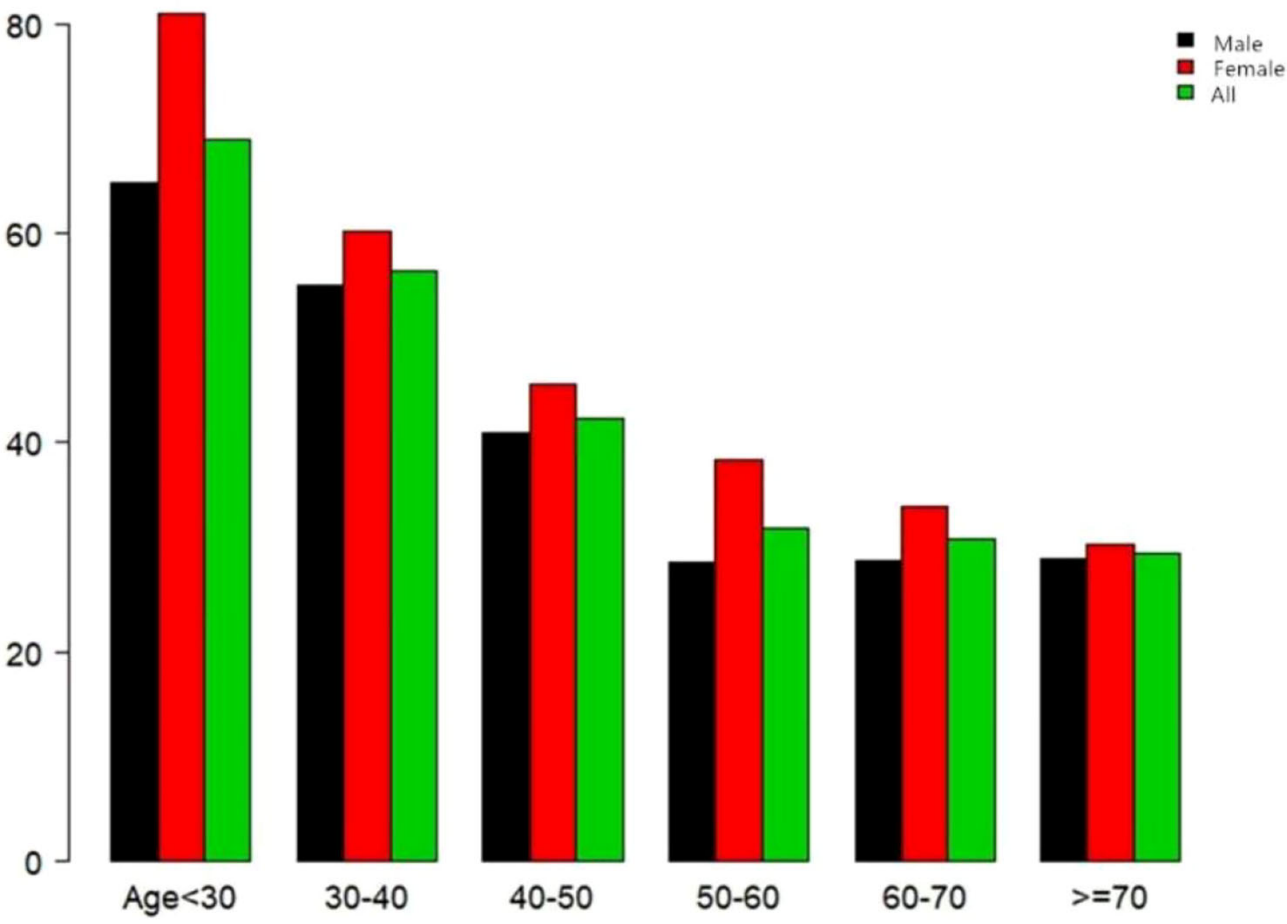
Rate of reversion to normoglycemia in patients with prediabetes stratified by age in 10-year intervals. The female participants with prediabetes showed a higher rate of reversion to normoglycemia than men, regardless of their age group. In addition, the reversion rates in both men and women decreased with increasing age.

In this study, 4,602 individuals reverted from IFG to normoglycemia, with a total cumulative rate of 13.8/100 person-years. Differences in the cumulative rate of reversion to normoglycemia were found between the four quartile groups, ranging from 12.9 to 16.4/100 person-years. Furthermore, the reversion rates in Q1–Q4 were as follows: 38.2 (36.4–40.0), 38.5 (36.7–40.3), 40.2 (38.3–42.0), and 48.5 (46.7–50.4). It was found that participants in the higher quartiles exhibited higher rates of reversion to normoglycemia (*p* < 0.0001 for trend) (**Table 2**, **Figure 3**). In **Figure 4**, the population was stratified by gender and age groups. Within each age group, women consistently showed higher reversion rates than men. As age increased, the reversion rates for both men and women decreased.

**Table 2:** Rate of reversion to normoglycemia in individuals with impaired fasting glucose (IFG).

AST/ALT ratio	Participants (*n*)	Reversion events (*n*)	Reversal rate (%) (95%CI)	Per 100 person-years
Total	11,121	4,602	41.4 (40.5–42.3)	13.8
Q1 (<0.81)	2,780	1,062	38.2 (36.4–40.0)	12.90
Q2 (0.81–1.08)	2,780	1,071	38.5 (36.7–40.3)	12.8
Q3 (1.08–1.40)	2,747	1,103	40.2 (38.3–42.0)	13.2
Q4 (>1.40)	2,814	1,366	48.5 (46.7–50.4)	16.4
*p* for trend			<0.001	

*AST/ALT ratio*, aspartate aminotransferase/alanine aminotransferase ratio; *CI*, confidence interval.

### Univariate analysis using a Cox proportional hazards regression model

**Table 3** shows the results of the univariate analysis used to explore the relationship between the reversion rate and several variables. As shown in the table, the reversion rate was not associated with BUN, ever drinkers, ever smokers, or those who had a family history of diabetes (*p* > 0.05). It was also found that the reversion rate was positively correlated with female gender, the AST/ALT ratio, HDL-c, and never smokers or drinkers, but was negatively correlated with older age, male gender, higher DBP and BMI, and increased FPG, cholesterol, SBP, TG, AST, and LDL-c levels.

**Table 3:** Factors influencing reversion to normoglycemia among participants with impaired fasting glucose (IFG) analyzed using univariate Cox proportional hazards regression.

Variable	Statistics	HR (95%CI)	*P*-value
Age (years)	49.90 ± 13.99	0.97 (0.97–0.98)	<0.0001
Gender			
Men	7,472 (67.19%)	Ref	
Women	3,649 (32.81%)	1.18 (1.11–1.25)	<0.0001
BMI (kg/m^2^)	24.85 ± 3.34	0.94 (0.93–0.95)	<0.0001
SBP (mmHg)	127.64 ± 17.70	0.99 (0.99–0.99)	<0.0001
DBP (mmHg)	78.46 ± 11.14	0.98 (0.98–0.99)	<0.0001
FPG (mmol/L)	5.95 ± 0.32	0.18 (0.16–0.20)	<0.0001
Cholesterol (mmol/L)	4.99 ± 0.95	0.89 (0.86–0.92)	<0.0001
Triglycerides (mmol/L)	1.78 ± 1.40	0.89 (0.86–0.91)	<0.0001
HDL-c (mmol/L)	1.34 ± 0.30	1.94 (1.80–2.10)	<0.0001
LDL-c (mmol/L)	2.89 ± 0.71	0.93 (0.89–0.97)	0.0004
ALT (U/L)	28.15 ± 22.27	1.00 (0.99–1.00)	<0.0001
AST (U/L)	26.34 ± 11.59	0.99 (0.99–0.99)	<0.0001
AST/ALT ratio	1.14 ± 0.43	1.32 (1.23–1.41)	<0.0001
BUN (mmol/L)	5.06 ± 1.27	0.98 (0.96–1.01)	0.1336
Scr (μmol/L)	74.09 ± 16.10	1.00 (1.00–1.00)	0.2087
Smoking status			
Current smoker	1,623 (14.59%)	Ref	
Ever smoker	378 (3.40%)	1.10 (0.93–1.31)	0.2721
Never smoker	9,120 (82.01%)	1.19 (1.10–1.30)	<0.0001
Drinking status			
Current drinker	481 (4.33%)	Ref	
Ever drinker	1,951 (17.54%)	1.12 (0.95, 1.32)	0.1813
Never drinker	8,689 (78.13%)	1.35 (1.16, 1.57)	0.0001
Family history of diabetes			
No	10,858 (97.64%)	Ref	
Yes	263 (2.36%)	0.96 (0.80, 1.16)	0.6803

*BMI*, body mass index; *FPG*, fasting plasma glucose; *LDL-c*, low-density lipoprotein cholesterol; *DBP*, diastolic blood pressure; *ALT*, alanine aminotransferase; *SBP*, systolic blood pressure; *AST*, aspartate aminotransferase; *non-HDL-c*, non-high-density lipoprotein cholesterol; *BUN*, blood urea nitrogen; *HDL-c*, high-density lipoprotein cholesterol; *Scr*, serum creatinine; *AST/ALT* ratio, aspartate aminotransferase/alanine aminotransferase ratio; *HR*, hazard ratio; *CI*, confidence interval; *Ref*, reference.

**Figure 5:**
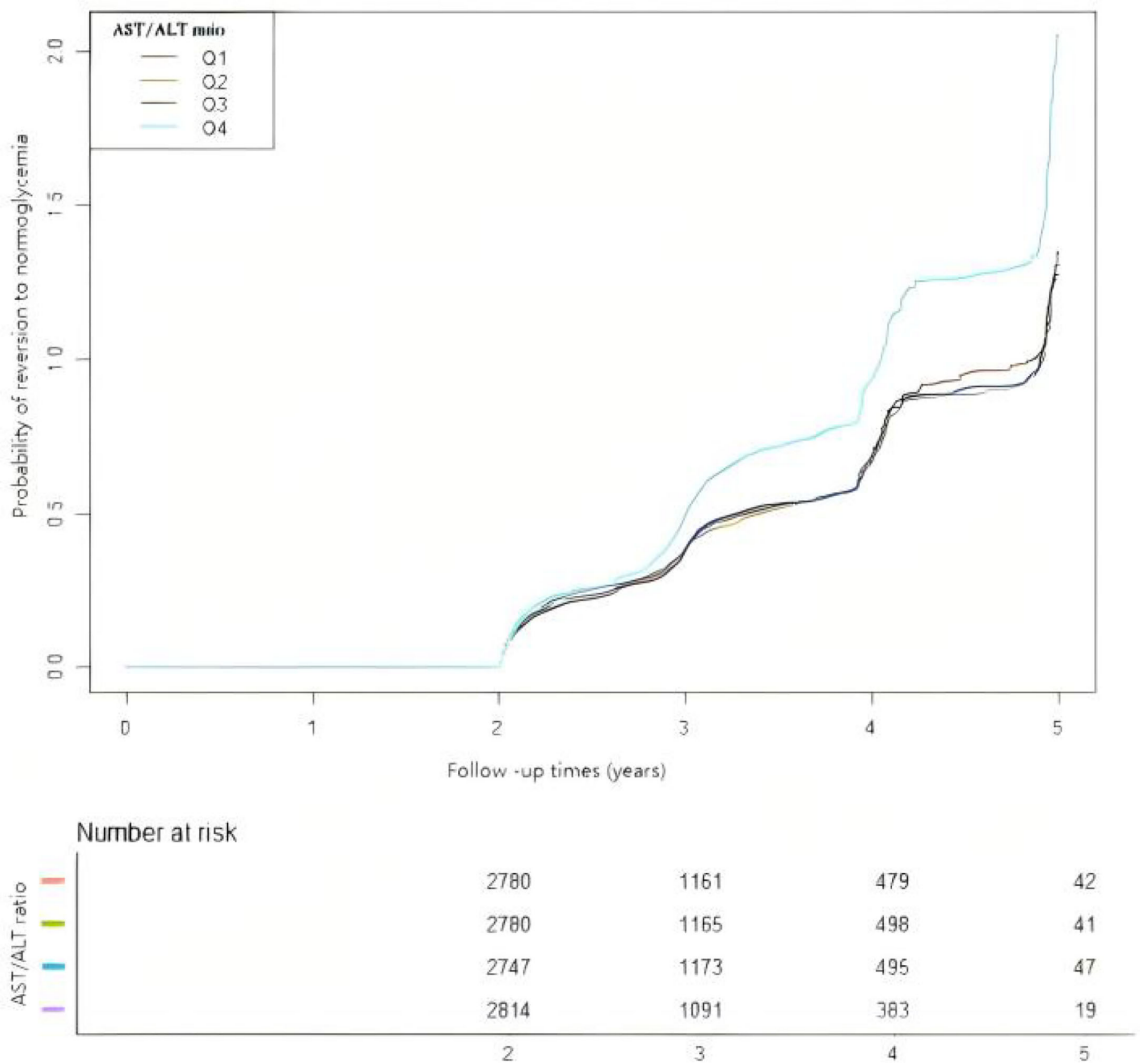
Kaplan–Meier curves for the probability of reversion to normoglycemia from prediabetes. The Kaplan–Meier curves were classified according to the quartiles of the aspartate aminotransferase (AST)/alanine transaminase (ALT) ratio to show the possibility of recovering from prediabetes to normoglycemia. The results showed that individuals with prediabetes in the highest AST/ALT ratio quartile have the highest chance of transitioning from prediabetes to normoglycemia.

In **Figure 5**, the Kaplan–Meier curves revealed the likelihood of reversion to normoglycemia from IFG according to the different AST/ALT ratio groups. There was a significant statistical effect on the AST/ALT ratio groups and the probability of reverting to normoglycemia (log-rank test: *p* < 0.001). Participants with higher AST/ALT ratios had higher chances of reverting to normoglycemia from IFG.

### Multivariate analysis using a Cox proportional hazards regression model

A Cox proportional-hazards regression model was used to illustrate the relationship between the AST/ALT ratio and the likelihood of reversion to normoglycemia (**Table 4**). A significant positive correlation between the AST/ALT ratio and the reversion to normoglycemia was discovered in model I. With every 1-unit increase in the AST/ALT ratio, the likelihood of blood glucose reverting to normoglycemia increased by 32% (HR = 1.32, 95%CI = 1.23–1.41). In model II, we adjusted for only the demographic variables, and the results also showed similar findings (HR = 1.33, 95%CI = 1.23–1.43). In model III (fully adjusted model), the positive correlation between the AST/ALT ratio and the reversion to normoglycemia could still be found (HR = 1.20, 95%CI = 1.11–1.30). All of the results were statistically significant (*p* < 0.05).

**Table 4:** Relationship between the aspartate aminotransferase (AST)/alanine aminotransferase (ALT) ratio and reversion to normoglycemia in subjects with impaired fasting glucose (IFG) in different models.

Exposure	Model I (HR, 95%CI,*p*)	Model II (HR, 95%CI,*p*)	Model III (HR, 95% CI,*p*)	Model IV (HR, 95%CI,*p*)
AST/ALT ratio	1.32 (1.23–1.41) <0.00001	1.33 (1.23–1.43) <0.00001	1.20 (1.11–1.30) <0.00001	1.16 (1.07–1.25) 0.00033
AST/ALT ratio				
Q1	Ref	Ref	Ref	Ref
Q2	0.98 (0.90–1.06) 0.58252	1.03 (0.95–1.13) 0.47361	0.98 (0.90–1.07) 0.60127	0.97 (0.89–1.06) 0.55157
Q3	0.98 (0.90–1.07) 0.67763	1.04 (0.95–1.13) 0.45471	0.96 (0.88–1.05) 0.40326	0.94 (0.86–1.03) 0.20906
Q4	1.30 (1.20–1.41) <0.00001	1.32(1.20–1.45) <0.00001	1.17 (1.07–1.29) 0.00100	1.13 (1.03–1.24) 0.01404
*p* for trend	<0.00001	<0.00001	0.00118	0.02112

Model I: Not adjusted for other covariates. Model II: Adjusted for gender, age, BMI, systolic blood pressure (SBP), diastolic blood pressure (DBP), family history of diabetes, and smoking and drinking status. Model III: Adjusted for age, gender, BMI, SBP, DBP, fasting plasma glucose (FPG), triglyceride, high-density lipoprotein cholesterol (HDL-c), low-density lipoprotein cholesterol (LDL-c), blood urea nitrogen (BUN), serum creatinine (Scr), family history of diabetes, and smoking and drinking status. Model IV: Adjusted for age (smooth), gender, BMI (smooth), SBP (smooth), DBP (smooth), FPG (smooth), triglycerides (smooth), HDL-c (smooth), LDL-c (smooth), BUN (smooth), Scr (smooth), family history of diabetes, and smoking and drinking status.

*HR*, hazard ratio; *CI*, confidence interval; *Ref*, reference.

### Results of the competing risk multivariate Cox proportional hazards regression

**Table 5** shows the competing risk analysis results from IFG to the incidence of diabetes. In model I (unadjusted model), it was discovered that, as the AST/ALT ratio increased, the incidence of reverting to normoglycemia also increased (HR = 1.32, 95%CI = 1.23–1.41). In model II, only the demographic variables were adjusted for. The results showed that the correlation between the AST/ALT ratio and the glucose reversion rate still remained positive (HR = 1.33, 95%CI = 1.23–1.43). In model III (fully adjusted model), the relationship still remained positive (HR = 1.20, 95%CI = 1.11–1.30). All of the results were statistically significant (*p* < 0.05).

**Table 5:** Relationship between the aspartate aminotransferase (AST)/alanine aminotransferase (ALT) ratio and the reversion to normoglycemia in subjects with impaired fasting glucose (IFG) in different models with competing risk of progression to diabetes.

Exposure	Model I (SHR, 95%CI,*p)*	Model II (SHR, 95%CI,*p*)	Model III (SHR, 95%CI,*p*)
AST/ALT ratio	1.32 (1.23–1.41) <0.00001	1.33 (1.23–1.43) <0.0001	1.20 (1.11–1.30) <0.0001
AST/ALT ratio			
Q1	Ref	Ref	Ref
Q2	0.98 (0.90–1.06) 0.5825	1.03 (0.95–1.13) 0.4736	0.98 (0.90–1.07) 0.6013
Q3	0.98 (0.90–1.07) 0.6776	1.04 (0.95–1.13) 0.4547	0.96 (0.88–1.05) 0.4033
Q4	1.30 (1.20–1.41) <0.00001	1.32 (1.20–1.45) <0.0001	1.17 (1.07–1.29)0.0010
*p* for trend	<0.00001	<0.0001	0.0012

Model I: Not adjusted for other covariates. Model II: Adjusted for gender, age, BMI, systolic blood pressure (SBP), diastolic blood pressure (DBP), family history of diabetes, and smoking and drinking status. Model III: Adjusted for age, gender, BMI, SBP, DBP, fasting plasma glucose (FPG), triglycerides, high-density lipoprotein cholesterol (HDL-c), low-density lipoprotein cholesterol (LDL-c), blood urea nitrogen (BUN), serum creatinine (Scr), family history of diabetes, and smoking and drinking status.SHR, subhazardratio.

### Sensitivity analysis

Several sensitivity analyses were conducted to prove the reliability of our findings. First, the AST/ALT ratio was treated as a continuous variable. Second, the AST/ALT ratios were partitioned into quartiles with equidistant patterns of effect sizes for each group and then were imported into the model. The results indicated that, in the unadjusted and the minimally adjusted models (model I and model II, respectively), the *p*-value of the trend was consistent with the results when the AST/ALT ratio was used as a continuous variable (shown in **Tables 4**, **5**).

A GAM was utilized to incorporate the continuous covariate as a curve in the equation (shown as model IV in **Table 4**). The results indicated that the AST/ALT ratio was positively related to the possibility of reverting to normoglycemia, showing an HR of 1.16 (95%CI = 1.07–1.25, *p* = 0.00033).

In addition, in **Table 6**, in order to exclude the effect of smoking or drinking habits or participants with a family history of diabetes, supplementary sensitivity analyses were conducted to improve our findings. Due to the missing data on smoking and alcohol consumption, almost reaching 70%, these variables were excluded as covariates in some of the sensitivity analyses. However, despite their exclusion, the findings were consistent with our previous results. The participants with no drinking status (**Table 6**, model a) showed a positive association between the AST/ALT ratio and the possibility of reverting to normoglycemia (HR = 1.19, 95%CI = 1.09–1.30). Participants with no smoking status (**Table 6**, model a) also presented a similar outcome (HR = 1.17, 95%CI = 1.08–1.28). After excluding participants with a family history of diabetes (**Table 6**, model c), the result still remained positive.

**Table 6:** Relationship between the aspartate aminotransferase (AST)/alanine aminotransferase (ALT) ratio and the probability of reverting from impaired fasting glucose (IFG) to normoglycemia in different sensitivity analyses.

Exposure	Model a (HR, 95%CI,*p*)	Model b (HR, 95%CI,*p*)	Model c (HR, 95%CI,*p*)	Model d (HR, 95%CI,*p*)
AST/ALT ratio	1.19 (1.09–1.30) <0.0001	1.17 (1.08–1.28) 0.0002	1.186 (1.097–1.283) 0.00002	1.198 (1.109–1.295) <0.00001
AST/ALT ratio				
Q1	Ref	Ref	Ref	Ref
Q2	0.97 (0.87–1.07) 0.5267	0.98 (0.89–1.08) 0.6469	0.964 (0.883–1.053) 0.41910	0.976 (0.894–1.065) 0.58445
Q3	0.94 (0.85–1.04) 0.2515	0.96 (0.87–1.06) 0.4411	0.952 (0.868–1.044) 0.29530	0.962 (0.878–1.055) 0.40918
Q4	1.14 (1.02–1.26) 0.0218	1.14 (1.02–1.26) 0.0156	1.157 (1.051–1.274) 0.00294	1.174 (1.068–1.292) 0.00093
*p* for trend	0.0157	0.0128	0.00299	0.00107

Model a was a sensitivity analysis performed on never-drinker participants (*n* = 8,689). We adjusted for age, gender, BMI, systolic blood pressure (SBP), diastolic blood pressure (DBP), fasting plasma glucose (FPG), triglyceride, high-density lipoprotein cholesterol (HDL-c), low-density lipoprotein cholesterol (LDL-c), blood urea nitrogen (BUN), serum creatinine (Scr), smoking status, and family history of diabetes. Model b was a sensitivity analysis performed on never-smoker participants (*n* = 9,120). We adjusted for gender, BMI, age, SBP, DBP, FPG, triglyceride, HDL-c, LDL-c, BUN, Scr, drinking status, and family history of diabetes, Model c was a sensitivity analysis in participants without a family history of diabetes (*n* = 10,858). We adjusted for age, BMI, SBP, gender, DBP, FPG, triglyceride, HDL-c, LDL-c, BUN, and Scr. Model d was a sensitivity analysis in participants without smoking and drinking status (*n* = 11,121). We adjusted for age, BMI, SBP, gender, DBP, FPG, triglycerides, HDL-c, LDL-c, BUN, Scr, and family history of diabetes.

*HR*, hazard ratio; *CI*, confidence interval; *Ref*, reference.

### Nonlinear relationship between the AST/ALT ratio and the incidence of glucose reversion

**Figure 6:**
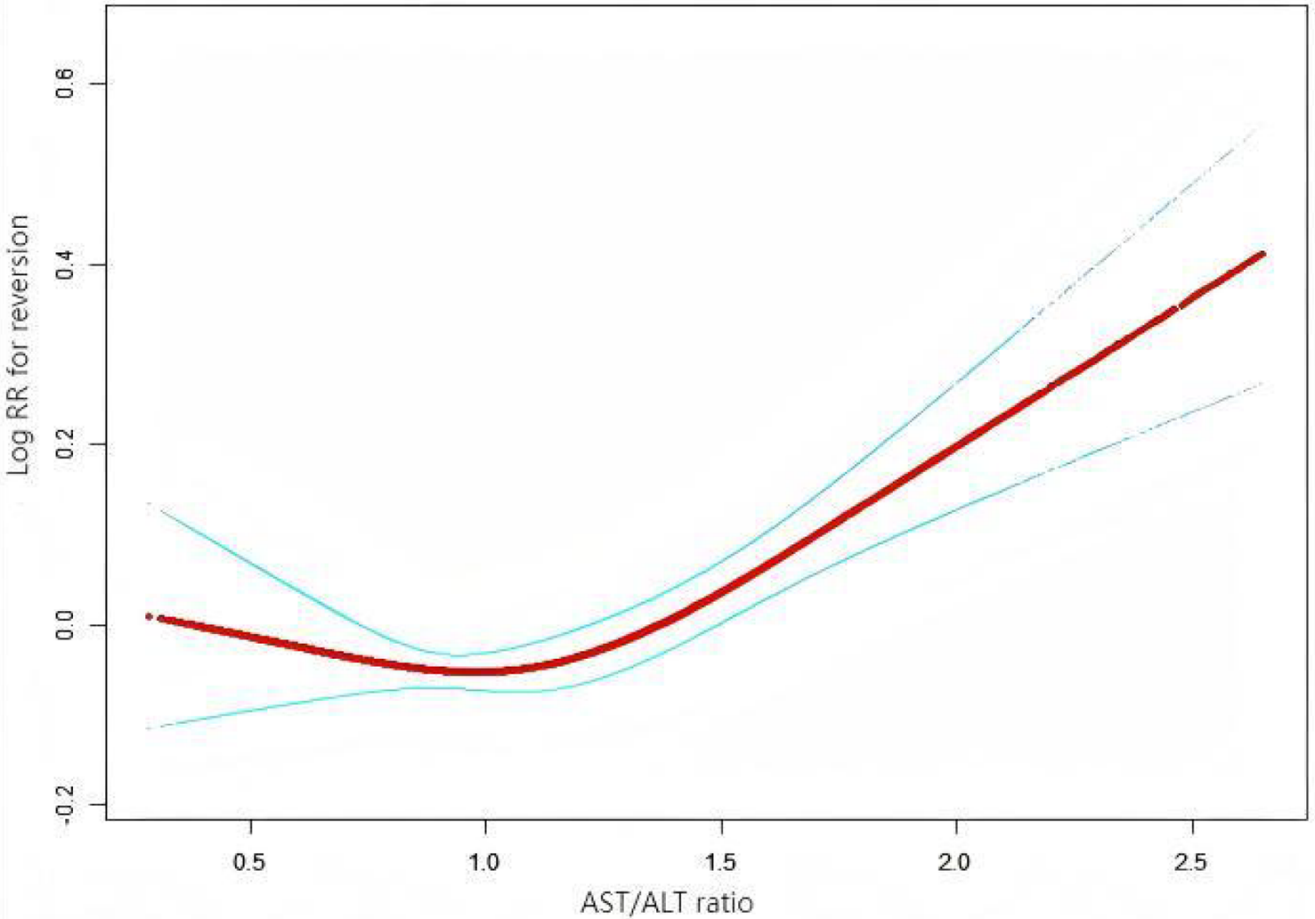
Nonlinear relationship between the aspartate aminotransferase (AST)/alanine transaminase (ALT) ratio and the reversion to normoglycemia in patients with prediabetes. We employed a Cox proportional hazards regression model with cubic spline functions to investigate the association between the AST/ALT ratio and the probability of reversion from prediabetes to normoglycemia. The findings revealed a nonlinear relationship between the AST/ALT ratio and this probability, with an inflection point observed at 1.13.

To further investigate the relationship between the AST/ALT ratio and the incidence of glucose reversion, a Cox proportional hazards regression model and a cubic spline function were applied (**Figure 6**). It was found that the relationship between the AST/ALT ratio and the glucose reversion rate is nonlinear. In order to find the best fit, a binary two-stage Cox proportional hazards regression model and a logarithmic likelihood ratio test were further applied (**Table 7**). The inflection point was determined to be 1.13. When the AST/ALT ratio is >1.13, it is positively associated with the incidence of glucose reversion (HR = 1.37, 95%CI = 1.23–1.52). However, with the AST/ALT ratio <1.13, their relationship is not significant.

**Table 7:** Results of the two-piecewise Cox regression model.

Probability of reversion to normoglycemia	HR (95%CI)	*P*-value
Fitting model by standard Cox regression	1.20 (1.11–1.30)	<0.0001
Fitting model by a two-piecewise Cox regression model		
Inflection point of the AST/ALT ratio	1.13	
≤Inflection point	0.90 (0.75–1.08)	0.2610
>Inflection point	1.37 (1.23–1.52)	<0.0001
*p* for log-likelihood ratio test		<0.001

We adjusted for gender, BMI, age, systolic blood pressure (SBP), diastolic blood pressure (DBP), fasting plasma glucose (FPG), triglycerides, high-density lipoprotein cholesterol (HDL-c), low-density lipoprotein cholesterol (LDL-c), blood urea nitrogen (BUN), serum creatinine (Scr), CCR, family history of diabetes, and smoking and drinking status.

*HR*, hazard ratio; *CI*, confidence interval.

### Results of the subgroup analyses

In order to investigate the association between the AST/ALT ratio and the likelihood of reverting to normoglycemia in different populations, the interactions between various variables in the prespecified subgroups and the exploratory subgroups were comprehensively evaluated (**Table 8**). It was found that there was no significant interaction between BMI, age, gender, SPB, DPB, or TG. However, it is worth noting that an interaction between the variables related to FPG was observed.

**Table 8:** Stratified associations between the aspartate aminotransferase (AST)/alanine aminotransferase (ALT) ratio and the reversion to normoglycemia in subjects with impaired fasting glucose (IFG) in the prespecified and exploratory subgroups.

Characteristic	No. of participants	HR (95%CI)	*P*-value	*P* for interaction
Age (years)				0.584
<45	4,318	1.13 (0.96–1.26)	0.030	
≥45	6,803	1.17(1.05–1.30)	0.003	
Gender				0.500
Men	7,472	1.22 (1.06–1.39)	0.004	
Women	3,649	1.25 (1.05–1.48)	0.013	
BMI (kg/m^2^)				0.249
<18.5	188	0.91 (0.56–1.47)	0.695	
≥18.5, <24	4,336	1.15 (1.04–1.28)	0.010	
≥24, <28	4,780	1.29 (1.13–1.47)	0.0001	
≥28	1,817	1.37 (1.06–1.77)	0.016	
SBP (mmHg)				0.076
<140	8,675	1.16 (1.07–1.26)	0.0005	
≥140	2,446	1.40 (1.16–1.68)	0.0005	
DBP (mmHg)				0.581
<90	9,484	1.21 (1.12–1.31)	<0.0001	
≥90	1,637	1.13 (0.89–1.44)	0.327	
FPG (mmol/L)				0.002
<6.1	8,121	1.18 (1.08–1.28)	<0.0001	
≥6.1	3,000	1.62 (1.34–1.96)	<0.0001	
Triglycerides (mmol/L)				0.092
<1.7	6,858	1.23 (1.13–1.35)	< 0.001	
≥1.7	4,263	1.07 (0.93–1.24)	0.351	

The above model was adjusted for gender, BMI, age, systolic blood pressure (SBP), diastolic blood pressure (DBP), triglycerides, high-density lipoprotein cholesterol (HDL-c), low-density lipoprotein cholesterol (LDL-c), alanine aminotransferase ratio (ALT), aspartate aminotransferase (AST), blood urea nitrogen (BUN), serum creatinine (Scr), family history of diabetes, and smoking and drinking status. In each case, the model was not adjusted for the stratification variable.

*HR*, hazard ratio; *CI*, confidence interval; *Ref*, reference.

Specifically, a more significant correlation between the AST/ALT ratio and the probability of returning to normal blood glucose levels was found in participants with FPG levels ≥6.1 mmol/L (HR = 1.62, 95%CI = 1.34–1.96). However, in participants with FPG levels <6.1 mmol/L, the association between the AST/ALT ratio and the likelihood of IFG returning to normal blood glucose levels was weaker (HR = 1.18, 95%CI = 1.08–1.28).

### Diagnostic value of the AST/ALT ratio in the reversion to normoglycemia in individuals with IFG

**Figure 7:**
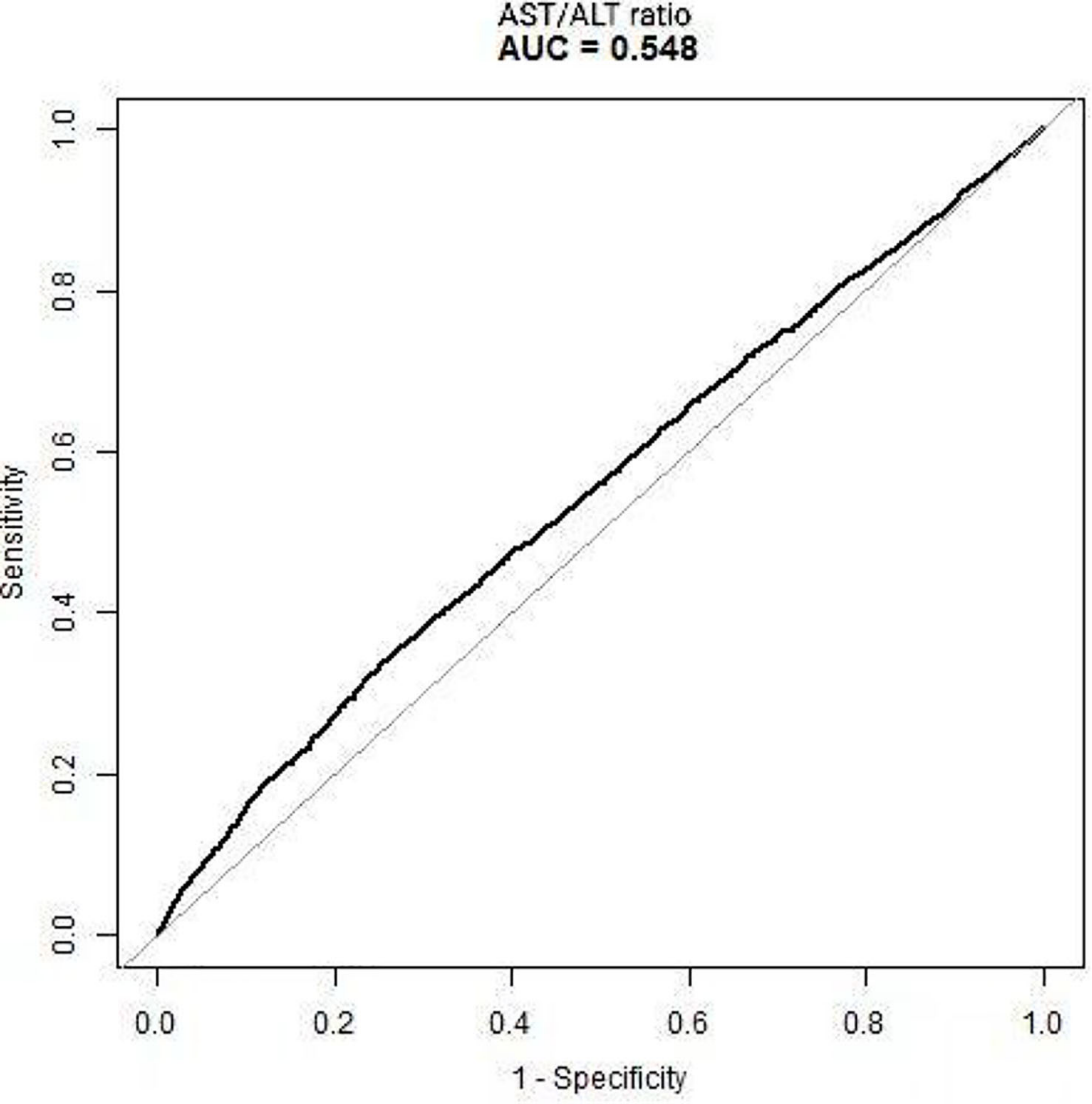
Receiver operating characteristic (ROC) curve of the aspartate aminotransferase (AST)/alanine transaminase (ALT) ratio for the reversion to normoglycemia in individuals with impaired fasting glucose (IFG). The diagnostic accuracy of the AST/ALT ratio in differentiating between participants with IFG with and without reversion to normoglycemia was analyzed using the ROC method. The AST/ALT ratio had an area under the curve (AUC) of 0.5480 (95%CI = 0.5371–0.5589).

The results above indicate that the AST/ALT ratio and the reversion to normoglycemia in individuals with IFG were independently associated. We further analyzed the diagnostic value of the AST/ALT ratio for the reversion to normoglycemia in individuals with IFG. The ROC method was applied to analyze the diagnostic accuracy of the AST/ALT ratio for detecting reversion to normoglycemia in individuals with IFG. The AST/ALT ratio had an AUC of 0.5480 (95%CI = 0.5371–0.5589). When the cutoff point of the AST/ALT ratio was set at 1.3176 to discriminate the reversion to normoglycemia, it would meet the highest Youden’s index (0.0837) and the diagnostic accuracy of sensitivity (35.25%), specificity (73.12%), and PPV (48.07%)/NPV (61.53%). As the NPV was 61.53%, there was a 61.53% chance that a patient with IFG and an AST/ALT ratio below 1.3176 would not revert to normoglycemia (**Table 9**, **Figure 7**).

**Table 9:** Diagnostic accuracy of the aspartate aminotransferase (AST)/alanine aminotransferase ratio (ALT) ratio for reversion to normoglycemia in subjects with impaired fasting glucose (IFG).

	AUC	95%CI low	95%CI high	Cutoff	SP (%)	SE (%)	PPV (%)	NPV (%)	Youden’s index
AST/ALT ratio	0.5480	0.5371	0.5589	1.3176	73.12	35.25	48.07	61.53	0.0837

*AUC*, area under the curve; *PPV*, positive predictive value; *SP*, specificity; *NPV*, negative predictive value; *SE*, sensitivity.

## Discussion

In this retrospective cohort study, we explored the relationship between the AST/ALT ratio and the likelihood of reverting to normoglycemia in patients with IFG. The findings demonstrated that 41.4% of the participants with IFG reverted to normoglycemia. Crucially, we discovered a nonlinear relationship between the AST/ALT ratio and the probability of reversion to normoglycemia in individuals with IFG. Furthermore, a threshold effect emerged, revealing differing relationships between the AST/ALT ratio and the reversion to normoglycemia across the inflection point.

Several other studies are in line with our findings. In this retrospective cohort study, we investigated the relationship between the AST/ALT ratio and the likelihood of reverting to normoglycemia among patients with IFG. The findings revealed that 41.4% of the participants with IFG reverted to normoglycemia. In a prospective cohort study conducted in China, which recruited 14,231 participants, it was found that 44.9% of the participants reverted to normoglycemia from prediabetes after 2 years of follow-up (41). Similarly, a prospective cohort study in Mexico observed that, after 2.5 years of follow-up, 22.6% of the patients with IFG reverted to normoglycemia, while 22.9% progressed to type 2 diabetes mellitus (T2DM). Notably, a substantial proportion of the participants with IFG experienced regression to normoglycemia. Therefore, identifying factors that can contribute to the reversion of IFG to normal glucose levels is crucial.

Many results suggest that the AST/ALT ratio is related to DM and NAFLD. A retrospective cohort study of 87,883 participants conducted in China found that the AST/ALT ratio was negatively associated with the risk of DM, and a threshold effect was discovered (42). Similarly, in a Japanese population, a negative correlation between the AST/ALT ratio and the incidence of DM was also found (6). Furthermore, in a retrospective study consisting of 75,204 Chinese adults, the AST/ALT ratio was negatively associated with the development of prediabetes (43). Moreover, the AST/ALT ratio was also associated with IR and DM (6, 11, 12, 42, 44).

The close interaction between DM, NAFLD, and IR has long been a topic of discussion (45, 46). As previously mentioned, the AST/ALT ratio is negatively associated with the development of prediabetes. notably, it is not surprising to find that participants with higher AST/ALT ratios are more likely to revert to normoglycemia. However, no previous study has elucidated the relationship between the AST/ALT ratio and the reversion to normoglycemia. To our knowledge, this study is the first to demonstrate a positive association between the AST/ALT ratio and reversion to normoglycemia from prediabetes. Furthermore, sensitivity analyses were used to further confirm the reliability of our results.

The exact mechanism involved in the relationship between the AST/ALT ratio and the reversion to normoglycemia is not well established. However, the AST/ALT ratio can affect glucose metabolism in several aspects. The AST/ALT ratio is a biomarker for NAFLD. Zou et al. discovered that the AST/ALT ratio is negatively associated with NAFLD (7). Several mechanisms could explain the role of NAFLD in glucose metabolism. The most popular theory for the pathogenesis of NAFLD is the multiple-hit theory, which includes liver fat accumulation and consequent lipotoxicity, increased oxidative stress, mitochondrial dysfunction, and increased endoplasmic reticulum stress (47). Insulin resistance (IR) and elevated hepatic free fatty acids (FFAs) (termed lipotoxicity) play a pivotal role in the pathogenesis of NAFLD. Multiple molecular pathways are implicated in this pathogenic process. IR promotes lipolysis, resulting in the accumulation of excessive fatty acids within the liver (48). The accumulation of FFAs in the liver activates the inflammatory process through the activation of NF-κB kinase beta (IKK-β), which further promotes the activation of pro-inflammatory cytokines, such as TNF-α and IL-6 (46). This inflammatory activation subsequently induces serine phosphorylation of the insulin receptor substrate (IRS), exacerbating IR. Sanyal et al. conducted a clinical trial to determine whether elevated hepatic FFAs are linked to increased oxidative stress and mitochondrial dysfunction, aggravating the inflammatory process and IR (49). Moreover, an excessive amount of fat can also accumulate in the pancreas, causing non-alcoholic fatty pancreas disease (NAFPD), triggering a series of biological reactions that eventually result in IR, which is crucial for DM development (46, 50). Finally, an animal study found that cyclic fatty acid monomers not only induce hepatic steatosis but are also associated with an increase in AST/ALT levels (51).

As mentioned above, there is substantial evidence demonstrating that excessive FFAs and IR play pivotal roles in the development of both NAFLD and DM. Several molecular mechanisms involved in the activation of inflammation and the progression to IR have been well established. IR triggers FFA accumulation, thereby inducing direct lipid toxicity and increasing oxidative stress, along with endoplasmic reticulum stress, in turn activating inflammation (52). Moreover, excessive FFAs activate the JNK-1 and PKC pathways, which subsequently downregulate IRS-1/2 signaling, thereby worsening IR (52). When adipose tissue fails to store FFAs, the excess fat accumulates in the liver and pancreas. This ectopic fat accumulation triggers inflammation and oxidative stress, ultimately causing liver injury and the development of IR.

This study has several strengths. First, this is the first study investigating the relationship between the AST/ALT ratio and the reversion from prediabetes to normal glucose levels in the Chinese population. Second, using Cox regression models, we discovered a nonlinear relationship between the AST/ALT ratio and the incidence of glucose reversion, which is an important finding of this study. Third, to deal with missing data, we adopted the multiple imputation approach to minimize bias while maximizing statistical power. Fourth, we conducted a series of sensitivity analyses to confirm the reliability of our findings.

There are some possible limitations to this study. First, the participants were all Chinese, and more investigations are needed to examine the reliability of our results in other genetic backgrounds. Second, IFG is only one of the diagnostic criteria for prediabetes. However, measurement of 2-h glucose and glycated hemoglobin was challenging in such a study cohort. Third, this study was based on a secondary analysis of published data, and variables that were not included in these data, such as waist circumference, cannot be adjusted for. Fourth, as a retrospective study, our research could merely establish an association between the AST/ALT ratio and the incidence of recurrence from prediabetes, not a causal link. Finally, this study only analyzed the AST/ALT ratio at baseline. In addition, the complex calculation of the index could be a major limitation for daily clinical use. We encourage future studies to address this limitation or to collaborate with other researchers to further investigate changes in the AST/ALT ratio over time and the incidence of reversion from prediabetes.

## Conclusion

Our research revealed a significant nonlinear relationship between the AST/ALT ratio and normoglycemia reversion in individuals with IFG, characterized by a distinct threshold effect. The inflection point was calculated at 1.13. This relationship differed markedly before and after this inflection point. After the inflection point, the AST/ALT ratio demonstrated a positive correlation with the probability of glucose reversion.

## Data Availability

Publicly available datasets were analyzed in this study. The data can be found here: https://doi.org/https://doi.org/10.5061/dryad.ft8750v.
