# Supplementary material for: Association between the aspartate aminotransferase-to-alanine aminotransferase ratio and the reversion to normoglycemia in people with impaired fasting glucose: a 5-year retrospective cohort study
Source: Front Endocrinol (Lausanne). 2025 Sep 25;16:1546202. doi: 10.3389/fendo.2025.1546202 (PMC12507602; doi:10.3389/fendo.2025.1546202)
Supplement: Supplementary file 1 [file Table1.docx]

**Association between aspartate aminotransferase to alanine aminotransferase ratio and reversion to normoglycemia in people with impaired fasting glucose: a 5-year retrospective cohort study**

**Running title: AST/ALT ratio and reversion to normoglycemia**

**Kebao Zhang,^1 #^  Lidan Chen,^2#^  Zhe Deng,^3^ Rong rong,^2^ Lifen Xu,^2^ Liting Xu, ^2^ Shuting Zeng ,^2^ Haofei Hu^4^***

^1^Department of Emergency, The Eighth Affiliated Hospital, Sun Yat-sen University, Shenzhen 518000, Guangdong Province, China

^2^Shenzhen Nanshan Medical Group Headquarter, Shenzhen 518000, Guangdong Province, China

^3^ Department of Emergency, Shenzhen Second People’s Hospital, Shenzhen 518000, Guangdong Province, China

^4^ Department of Nephrology, Shenzhen Second People’s Hospital, Shenzhen 518000, Guangdong Province, China

*Corresponding author

Haofei Hu

Department of Nephrology,

Shenzhen Second People’s Hospital

No.3002 Sungang Road, Futian District,

Shenzhen 518000,

Guangdong Province,

China

Tel:+86-755-83366388

1. mail: [huhaofei0319@126.com](mailto:huhaofei0319@126.com)

**Table S1. Collinearity diagnostics steps.**

| Variable | VIF  Step 1 |
| --- | --- |
|  |  |
| Gender | 1.9 |
| Age(years) | 1.2 |
| Smoking status | 1.2 |
| Drinking status | 1.2 |
| Family history of diabetes | 1.0 |
| HDL-c(mmol/L) | 1.2 |
| LDL-c(mmol/L) | 1.1 |
| BUN(mmol/L) | 1.1 |
| FPG(mmol/L) | 1.1 |
| Scr (umol/L) | 1.7 |
| BMI (kg/m^2^) | 1.2 |
| SBP (mmHg) | 1.8 |
| DBP (mmHg) | 1.7 |
| Cholesterol(mmol/L) | NA |
| Triglyceride(mmol/L) | 1.2 |

BMI, body mass index; FPG, fasting plasma glucose; DBP, diastolic blood pressure; TC, total cholesterol; SBP, systolic blood pressure; TG, triglyceride; ALT, alanine aminotransferase; LDL-c, low-density lipoprotein cholesterol; AST, aspartate aminotransferase; HDL-c, high-density lipoprotein cholesterol; BUN, blood urea nitrogen; Scr, serum creatinine.

Abbreviation: VIF: variance inflation factor; VIF = 1/(1-R^2^).

Note: The variables with VIF>5 will be regarded as collinear variables and cannot be included in the multiple regression model.

**Table S2. Relationship between gender and reversion to normoglycemia in people with IFG in different models**

| Exposure | (HR, 95% CI, P) (HR, 95% CI, P) | |
| --- | --- | --- |
| Gender |  |  |
| Male | 1.0 | 1.0 |
| Female | 1.18 (1.11, 1.25) <0.0001 | 1.07 (0.98, 1.17) 0.1120 |

we adjust age; SBP; DBP; FPG; triglyceride; HDL-c; LDL-c; ALT; AST; BUN; SCR; family history of diabetes, smoking and drinking status; BMI; ALT; AST

**Table S3. Relationship between BMI and reversion to normoglycemia in people with IFG in different models**

| Exposure | Model I (HR, 95% CI, P) | Model II (HR, 95% CI, P) |
| --- | --- | --- |
| BMI | 0.94 (0.93, 0.95) <0.0001 | 0.99 (0.98, 1.00) 0.0054 |
| BMI quartiles |  |  |
| <18.5 | 1.0 | 1.0 |
| >=18.5, <24 | 0.74 (0.62, 0.89) 0.0014 | 0.95 (0.79, 1.14) 0.5616 |
| >=24, <28 | 0.55 (0.45, 0.66) <0.0001 | 0.89 (0.74, 1.08) 0.2378 |
| >=28 | 0.47 (0.39, 0.58) <0.0001 | 0.86 (0.70, 1.06) 0.1513 |
| P for trend | <0.0001 | 0.0234 |

Model I: we did not adjust other covariates

Model II: we adjust age; gender; SBP; DBP; FPG; triglyceride; HDL-c; LDL-c; ALT; AST; BUN; SCR; family history of diabetes, smoking and drinking status

**Table S4. Relationship between DBP and reversion to normoglycemia in people with IFG in different models**

| Exposure | Model I (HR, 95% CI, P) | Model II (HR, 95% CI, P) |
| --- | --- | --- |
| DBP | 0.984 (0.982, 0.987) <0.00001 | 0.995 (0.991, 0.998) 0.00355 |
| DBP binary groups |  |  |
| <90 | 1.0 | 1.0 |
| >=90 | 0.674 (0.614, 0.740) <0.00001 | 0.877 (0.791, 0.972) 0.01280 |
| P for trend | <0.00001 | 0.01280 |

Model I: we did not adjust other covariates

Model II: we adjust age; gender; SBP; FPG; triglyceride; HDL-c; LDL-c; ALT; AST; BUN; SCR; family history of diabetes, smoking and drinking status;BMI

**Table S5. Relationship between SBP and reversion to normoglycemia in people with IFG in different models**

| Exposure | Model I (HR, 95% CI, P) | Model II (HR, 95% CI, P) |
| --- | --- | --- |
| SBP | 0.99 (0.99, 0.99) <0.0001 | 1.00 (1.00, 1.00) 0.9065 |
| SBP binary groups |  |  |
| <140 | 1.0 | 1.0 |
| >=140 | 0.66 (0.61, 0.71) <0.0001 | 0.95 (0.87, 1.04) 0.2573 |
| P for trend | 0.66 (0.61, 0.71) <0.0001 | 0.95 (0.87, 1.04) 0.2573 |

Model I: we did not adjust other covariates

Model II: we adjust age; gender; DBP; FPG; triglyceride; HDL-c; LDL-c; ALT; AST; BUN; SCR; family history of diabetes, smoking and drinking status;BMI
